# Characterization of stress response in human retinal epithelial cells

**DOI:** 10.1111/j.1582-4934.2012.01652.x

**Published:** 2012-12-04

**Authors:** Vincenzo Giansanti, Gloria E Villalpando Rodriguez, Michelle Savoldelli, Roberta Gioia, Antonella Forlino, Giuliano Mazzini, Marzia Pennati, Nadia Zaffaroni, Anna Ivana Scovassi, Alicia Torriglia

**Affiliations:** a Istituto di Genetica Molecolare, CNRPavia, Italy; b U872 eq. 17, Centre de Recherches des Cordeliers, INSERMParis, France; c Centre de Recherches des Cordeliers, Université Pierre et Marie CurieParis, France; d Centre de Recherches des Cordeliers, Université Paris DescartesParis, France; e Departement Ophtalmologie, Hotel DieuParis, France; f Dipartimento di Biochimica, Università di PaviaPavia, Italy; g Department of Experimental Oncology, Fondazione IRCCS Istituto Nazionale dei TumoriMilano, Italy

**Keywords:** Apoptosis, ARPE-19 cells, autophagy, caspases, HMA, L-DNase II, PARP-1

## Abstract

The pathogenesis of age-related macular degeneration (AMD) involves demise of the retinal pigment epithelium and death of photoreceptors. In this article, we investigated the response of human adult retinal pigmented epithelial (ARPE-19) cells to 5-(*N*,*N*-hexamethylene)amiloride (HMA), an inhibitor of Na^+^/H^+^ exchangers. We observed that ARPE-19 cells treated with HMA are unable to activate ‘classical’ apoptosis but they succeed to activate autophagy. In the first 2 hrs of HMA exposure, autophagy is efficient in protecting cells from death. Thereafter, autophagy is impaired, as indicated by p62 accumulation, and this protective mechanism becomes the executioner of cell death. This switch in autophagy property as a function of time for a single stimulus is here shown for the first time. The activation of autophagy was observed, at a lesser extent, with etoposide, suggesting that this event might be a general response of ARPE cells to stress and the most important pathway involved in cell resistance to adverse conditions and toxic stimuli.

## Introduction

Age-related macular degeneration (AMD) is the major cause of blindness in the Western countries. Moreover, the number of patients is expected to increase from 1.75 million to 3 million in the next 10 years [1]. The pathogenesis of AMD involves demise of the retinal pigment epithelium (RPE) followed by death of photoreceptors [2]. The RPE is a specialized epithelium at the interface between the neural retina and the choriocapillaris where it forms the outer blood–retinal barrier. RPE daily phagocytes important amounts of lipid-rich material (photoreceptor outer segment) in the most oxygenated part of the body. So that, these cells possess high levels of the enzymes required to detoxify reactive oxygen species (ROS) [3]. Because elevated ROS formation is involved in the pathogenesis of many diseases, including cancer, diabetes and neurodegenerative diseases, as well as in ageing, the discovery of biochemical mechanisms that either protect cells or promote cellular recovery from damage is extremely important.

In the intact eye, the transition from light to dark alters pH, [Ca^2+^] and [K^+^], and the oxygen consumption increases inducing the release of CO_2_ and H_2_O [4]. The RPE maintains pH and volume homeostasis of the subretinal space by transporting these products to the choroidal compartment. This manoeuvre decreases intracellular pH. In altered metabolic states like hypoxia and hyperglycaemia, H^+^ accumulates because of the elevated glycolysis and failure of retinal circulation, thus the retina is readily acidified, affecting thus the intracellular pH of the surrounding cells. It is worth noting that a decrease in intracellular pH is also a common effect of many apoptotic stimuli such as cytokine deprivation or ROS generation [5–7].

To study the biochemistry and molecular biology of oxidative stress in RPE cells, many investigators have used *in vitro* cultures of ARPE-19 cells. These cells have spontaneously arise from a human RPE cell line derived from the normal eyes of a 19-year-old male who died from head trauma in a motor vehicle accident [8]. ARPE-19 cells presenting a normal karyotype are able to form polarized epithelial monolayers and express RPE-specific markers such as CRALBP and RPE-65.

In this article, we investigated the response of human adult retinal pigmented epithelial (ARPE-19) cells to 5-(*N*,*N*-hexamethylene)amiloride (HMA), an inhibitor of Na^+^/H^+^ exchangers, *i.e*. a class of proteins acting as intracellular pH regulators [7], and we evaluated the pathways activated by these cells to face this metabolic stress. We focused on the analysis of apoptosis (both caspase-dependent and -independent), parthanatos and autophagy.

We observed that ARPE-19 cells treated with HMA are unable to drive ‘classical’ apoptosis and succeed in activating autophagy, which protects them under mild stress conditions, and ultimately leads to cell death.

## Materials and methods

### Cell culture

Human ARPE-19 cells (adult retinal pigmented epithelial) and HeLa cells (from human cervix carcinoma) were grown as monolayer at 37°C in humidified atmosphere containing 5% CO_2_. ARPE-19 cells were cultured in D-MEM/F12 (1:1) supplemented with 10% FCS, 4 mM glutamine, 100 U/ml penicillin and 0.1 mg/ml streptomycin; HeLa cells were cultured in D-MEM supplemented with 10% FCS, 4 mM glutamine, 2 mM Na/pyruvate, 100 U/ml penicillin and 0.1 mg/ml streptomycin (all reagents were from Celbio, Milano, Italy). Cells were cultured in 75 cm^2^ flasks and trypsinized when subconfluent.

### Cell treatments

ARPE-19 cells were treated with 5-(*N*,*N*-hexamethylene)amiloride (HMA; stock solution: 80 mM in DMSO, A9561; Sigma-Aldrich, Saint-Quentin Fallavier, France) at increasing concentrations (from 20 to 120 μM) for up to 72 hrs. As a positive control of apoptosis, cell cultures were treated with the DNA topoisomerase II inhibitor, etoposide, which is used as an internal standard for cell proliferation and viability assays. The 50 mM stock solution of etoposide (E1383; Sigma-Aldrich) was prepared in DMSO; ARPE-19 cells were treated with 250 μM etoposide for up to 72 hrs. In some experiments, HeLa cells treated with 100 μM etoposide for 3 hrs, followed by 24 hrs of recovery in drug-free medium were used as a positive example of apoptosis. As a positive control of autophagy, cell cultures were treated with 1 μM rapamycin (R0395; Sigma-Aldrich), an inhibitor of mTOR complex, for 2 hrs. Stock solution was prepared at the concentration of 1.367 mM in DMSO. To inhibit autophagy, 3-methyladenine (3-MA)(M9281; Sigma-Aldrich) and bafilomycin (B1793; Sigma-Aldrich) were used; the first one (stock solution 1 M) was administered at 5 mM for 4 hrs before the treatment with HMA and the second one at 50 nM (stock solution 8 μM in DMSO) for 2 hrs.

### Morphological analysis

For the staining with the fluorescent dye 4′,6-diamidino-2-phenylindole (DAPI), cells were seeded in 1 ml of complete medium at a density of 5 × 10^4^ cells/well in multiwells containing a coverslip (16-mm diameter) and treated 48 hrs later with HMA at the indicated concentrations. Multiwells with cells still attached to coverslips were put on a bed of ice, washed with cold PBS and fixed with 4% paraformaldehyde for 15 min. After the fixation, cells were permeabilized with 0.3% Triton in PBS for 30 min. at r.t., washed three times with PBS and stained with DAPI (0.2 μg/ml in PBS) for 10 min. in the dark. After five washings of 5 min. with PBS, slides were finally mounted with antifade mounting medium (90% glycerol, 20 mM Tris-HCl, pH 7.5, 2.33% DABCO). Cells were observed using a fluorescence microscope Olympus BX51, equipped with a 40x objective. The images were acquired through a digital camera Camedia C4040 (Olympus, Tokyo, Japan), Adobe Photoshop (Redwood City, CA, USA) was used as elaborating software.

### Transmission electron microscopy

ARPE-19 cells were seeded on Lab Tek slides, treated 48 hrs later with HMA at the indicated concentration, for 24 hrs. At the end of the treatment, cells were washed with PBS and fixed with 2.5% glutaraldehyde in sodium cacodylate for 30 min. and then washed in the same buffer twice for 15 min. Cells were post-fixed with 1% osmium tetroxide for 15 min. at 4°C in the dark, washed twice in cacodylate buffer and dehydrated in ethanol at increasing concentrations (50%, 70%, 80%, 90% and 100%). Slides were then incubated with Hydroxypropyl Methacrylate (64180; Sigma-Aldrich) twice for 5 min. each and embedded in epoxy resin. Ultrathin sections (80 nm) were contrasted by using uranyl acetate and observed using an electron microscope (model 100 CX II; JEOL, Tokyo, Japan).

### Cytotoxicity assay

Cytotoxicity assay was performed as already described [9].

### MTT assay

ARPE-19 cells were seeded in 96-multiwell plates at a density of 2.5 × 10^3^ in 100 μl of complete medium. Cells were treated 48 hrs later with different drug concentrations for 24–72 hrs. At the end of the treatment, 20 μl of CellTiter 96 non-radioactive cell proliferation assay (MTT, G4000; Promega, Milano, Italy) were added to each well. Plates were then incubated for 4 hrs at 37°C in the dark and analysed using a microplate reader (Gio De Vita, Rome, Italy) at 570/630 nm. Experiments were performed in quadruplicate and repeated three times.

### Indirect immunofluorescence experiments

ARPE-19 cells were seeded at the density of 5 × 10^4^/ml in 1 ml of complete medium in multiwells containing 16-mm diameter coverslips. Forty-eight hours later, cells were treated with HMA, etoposide, rapamycin or bafilomycin at the indicated concentrations. The following proteins: AIF, LAMP 2, LC3, LEI/L-DNase II, mtHSP70, p62/SQSTM1 and poly(ADP-ribose) were tested (see in Table 1 the list of the antibodies used).

**Table 1:** List of primary antibodies

Antigen	Primary antibody	Dilution	Company	Assay
AIF	4642	1:100	Cell Signaling	
Atg7	2631S	1:1000	Cell Signaling	WB
Atg5/12	2630	1:1000	Cell Signaling	WB
β-Actin	ab8229		Abcam	WB
Beclin-1	3738	1:1000	Cell Signaling	WB
Cleaved caspase-8	9496	1:1000	Cell Signaling	WB
Caspase-3	ALX-210-807	1:250	Alexis	WB
Cleaved caspase-9	9501	1:1000	Cell Signaling	WB
ERK1/2	442675	1:1000	Calbiochem	WB
^*^ERK1/2	442685	1:5000	Calbiochem	WB
JNK	559304	1:2000	Calbiochem	WB
^*^JNK	9251	1:2000	Cell Signaling	WB
Lamp 2	PRS 3627	1/100	Sigma-Aldrich	IF
LC3	2775	1:1000/1:100	Cell Signaling	WB/IF
mtHSP70	ALX-804-077	1:50	Alexis	IF
PARP-1	ALX-210-220-R100	1:1000	Alexis	WB
γ-Tubulin	T5326	1:10000	Sigma-Aldrich	WB
LEI/L-DNase II	Home-made	1:1000/		WB/IF
p62/SQSTM1	7695	1:10000/1:2000	Cell Signaling	WB/IF
PAR	ALX-804-220-R100	1:100	Alexis	IF
Survivin	ab8228	1/100	Abcam	WB
Thr^34^ Survivin	ab10720	1/1000	Abcam	WB

#### LC3

At the end of the treatment, cells were washed three times with PBS for 5 min., fixed with 4% paraformaldehyde for 15 min. on ice, washed three times in PBS, permeabilized with cold 100% acetone for 5 min., washed three times with PBS for 5 min. and then saturated with 4% BSA (bovine serum albumin) in PBS for 10 min. Slides were then incubated with the polyclonal antibody against LC3 (2775; Cell Signaling, Danvers, MA, USA) diluted 1:100 in PBS, for 1 hr at 37°C in a humidified chamber, washed five times with PBS, incubated with the secondary antibody (111-225-003, Cy2-conjugated anti-rabbit; Jackson, Suffolk, UK) diluted 1:50 in PBS, for 1 hr at 37°C in a humidified chamber. Coverslips were then washed three times with PBS in the dark and finally mounted with a drop of PBS.

#### LEI/L-DNase II and LAMP2

ARPE-19 cells labelling with LEI/L-DNase II antibodies was performed as described before [7].

#### mtHSP70/AIF and p62/SQSTM1

After fixation performed as above described, cells were kept overnight in 70% ethanol at −20°C. The day after, coverslips were washed three times in PBS, permeabilized with 0.3% Triton in PBS for 30 min. at r.t., washed three times with PBS and then saturated with 5% skim milk in PBS for 30 min. Slides were then incubated with the monoclonal antibody JG1 against mtHSP70 (ALX-804-077; Alexis, Villeurbanne, France), the polyclonal antibody against AIF (4642; Cell Signaling) and the polyclonal antibody against p62 (5114; Cell Signaling), diluted 1:50, 1:100 and 1:1000 in PBS, respectively, for 1 hr at 37°C in a humidified chamber, washed five times with PBS, incubated with a secondary antibody TRITC-conjugated anti-mouse (115-025-146; Jackson ImmunoResearch), Cy2-conjugated anti-rabbit (111-225-003; Jackson ImmunoResearch), and PerCP-conjugated anti-rabbit (111-126-144; Jackson ImmunoResearch) diluted 1:50 in PBS, for 1 hr at 37°C in a humidified chamber. Slides were then washed three times with PBS in the dark and finally mounted with a drop of PBS.

#### Poly(ADP-ribose)

After fixation performed as described above, cells were permeabilized with 0.3% Triton in PBS for 30 min. at r.t., washed three times with PBS and then saturated with 5% skim milk in PBS for 30 min. Coverslips were then incubated with the monoclonal antibody 10H against poly(ADP-ribose, ALX-804-220-R100; Enzo Lab Sciences, Lausen, Switzerland) diluted 1:100 in PBS, for 1 hr at 37°C in a humidified chamber, washed five times for 5 min. with PBS, incubated with the secondary antibody TRITC-conjugated anti-mouse (115-025-146; Jackson ImmunoResearch) diluted 1:50 in PBS, for 1 hr at 37°C in a humidified chamber. Coverslips were then washed three times with PBS in the dark and finally mounted with a drop of PBS. Cells were observed using a fluorescence microscope Olympus BX51, equipped with a 40x objective. The images were acquired with a digital camera Camedia C4040 (Olympus), Adobe Photoshop was used as elaborating software.

### Western blot analysis

Samples of 2.5 × 10^6^ cells (fresh or stored in liquid nitrogen) were resuspended with 100 μl of lysis buffer (10 mM Tris-HCl pH 7.6, 5 mM EDTA pH 8.0, 140 mM NaCl, 0.5% NP40), supplemented just before use with 2% protease inhibitor (RG7128; Hoffmann-La Roche, Milano, Italy) and 1.6 mM Na_3_VO_4_. Extracts were then heated for 10 min. either at 65°C (for PARP-1 and p62) or 95°C and run on denaturing polyacrylamide gel (12% for ATG7, ATG5/12, Beclin-1, cleaved caspase-9, ERK1/2, *ERK1/2, JNK, *JNK, PARP-1, p62/SQSTM1, γ-Tubulin; 12% for caspase-3, cleaved caspase-8, LC3, LEI/L-DNase II). Protein transfer was performed at 200 mA for 3 hrs at 4°C and monitored by the membrane staining with Red Ponceau (P3504; Sigma-Aldrich). The membrane was saturated with 5% skim milk in PBS for 1 hr at room temperature and incubated overnight at 4°C with the primary antibodies against Atg7 (1:1000; Cell Signaling), 2631S Atg5/12 (1:1000) (2630; Cell Signaling), Beclin-1 (1:1000) (3738; Cell Signaling), cleaved caspase-8 (1:1000) (9496; Cell Signaling), cleaved caspase-9 (1:1000) (9501; Cell Signaling), *JNK (1:2000) (9251; Cell Signaling), LC3 (1:1000) (2775; Cell Signaling), p62/SQSTM1 (1:10000) (7695; Cell Signaling), ERK1/2 (1:1000) (442675; Calbiochem, Darmstadt, Germany), *ERK1/2 (1:5000) (442685; Calbiochem), JNK (1:2000) (559304; Calbiochem), caspase-3 (1:250) (ADI-AAP-113-D; Alexis) and PARP-1 (1:1000) (ALX-210-220-R100; Alexis), γ-Tubulin (1:10000) (T5326; Sigma-Aldrich), LEI/L-DNase II (1:1000). After five washings in PBS containing 0.1% Tween-20, the membrane was incubated for 30 min. with the antimouse (115-035-174; Jackson ImmunoResearch) or anti-rabbit [(112-035-175; Jackson ImmunoResearch) secondary antibodies conjugated with horseradish peroxidase, diluted 1:10000 and 1:5000, in PBS containing 5% Skim Milk respectively] and then washed five times in PBS. Visualization of the immunoreactive bands was obtained by a chemoluminescent substrate, SuperSignal West Pico Chemiluminescent Substrate (Pierce 34087), or Immun-StarTM WesternCTM kit (170-5070; Bio-Rad, Segrate, Italy). Three independent experiments were carried out. To make the reading of the figures easier a table displaying all the antibodies used and their concentration has been included (Table 1).

### Analysis of survivin protein expression and phosphorylation

Cells were lysed with a specific cell extraction buffer (0.01% NP40, 10 mM Tris-HCl pH 7.5, 50 mM KCl, 5 mM MgCl_2_, 2 mM DTT, 20% glycerol, 1 mM PMSF plus protease and phosphatase inhibitors). For the assessment of the Thr^34^-phosphorylated form of survivin, precleared, detergent-solubilized cell extracts (100 μl) were immunoprecipitated with the rabbit polyclonal anti-human survivin antibody (ab8228; Abcam) for 16 hrs at 4°C, with precipitation of the immune complex by addition of 50 μl of a 50:50 protein A slurry (17-0963-03; GE Healthcare, Limonest, France). Samples were separated on precast 12% NuPAGE™ Bis-Tris gel (NP0321PK2; Invitrogen Life Technology, Merisiers, Saint Aubin, France) and transferred onto Hybond ECL nitrocellulose membranes (RPN203D; GE Healthcare) using the NuPAGE transfer buffer (NP0006; Invitrogen Life Technology). Nitrocellulose membranes were blocked in PBS-Tween 20 with 5% skim milk, first incubated with the primary antibodies specific for survivin and Thr^34^-phosphorylated survivin (ab10720; Abcam.) and then with the secondary peroxidase-conjugated antibodies. Bound antibodies were detected using the SuperSignals West PICO chemiluminescent substrate (34077; Pierce Biotechnology, Courtaboeuf, France) and autoradiography. β-Actin monoclonal antibody (ab8229; Abcam) was used to confirm equal protein loading on the gel. Results were quantified by densitometric analysis using the Image-Quant software (Molecular Dynamics, Sunnyvale, CA, USA).

### Internucleosomal DNA degradation

Internucleosomal DNA degradation assay was performed as already described [9].

### RNA extraction and real-time PCR

Total RNA was isolated from ARPE-19 using Qiazol Lysis Reagent (79306; Qiagen, Milano, Italy) according to the manufacturer's protocol. DNase treatment to eliminate DNA contaminations was performed using the Turbo DNA Free Kit (AM1907; Ambion, Monza, Italy). Total RNA concentration was determined spectrophotometrically and its quality was verified using the Bioanalyser 2001 (Agilent Technologies, Cernusco sul Naviglio, Italy). cDNA was synthesized starting from 1 μg of total RNA using the high capacity cDNA reverse transcription kit (4368814; Applied Biosystems, Monza, Italy) in a final volume of 50 μl. Real-time PCR reactions were run in a final volume of 25 μl on the Mx3000P Stratagene thermocycler using commercially available TaqMan primers and probes (Applied Biosystems) and TaqMan Universal PCR Master Mix (Applied Biosystems). All reactions were performed in triplicate. Expression levels for *Atg5* (Hs00169468_m1) and *Becn1* (Hs00186838_m1) were evaluated. *Gapdh* (Hs99999905_m1) was used as normalizer. Relative expression levels for each gene were calculated using the ΔΔCt method.

### Clonogenic Test

ARPE-19 cells were seeded in 1 ml of complete medium at the density of 2.3 × 10^4^ cells/well in 24-well plate and treated 48 hrs later with 80 μM HMA alone or in combination with 5 mM 3-MA or 1 μM rapamycin, as before. At the end of the treatment, cells were washed with PBS and trypsinized. For each condition, 10^3^ cells were seeded into 6-well plate and grown with complete medium. One week later, medium was removed and cells were carefully washed with PBS, fixed and stained with 2 ml of 6% glutaraldehyde and 0.5% cresyl violet. After 30 min., the staining solution was removed; plates were washed with tap water, and dried in normal air at room temperature. The surface of the plate occupied by the colonies was calculated as follows: pictures of each plate were taken in constant conditions of distance between the camera and the plate, source of light, ISO, zoom, exposition time and aperture of diaphragm. The area of analysis for the wells on the plate was selected (the radius was the same for all the wells). Using image analysis software, every image was binarized and the rate between the area occupied by colonies and the total area of the well was calculated.

## Results

### ARPE-19 cell response to HMA treatment

**Fig. 1:**
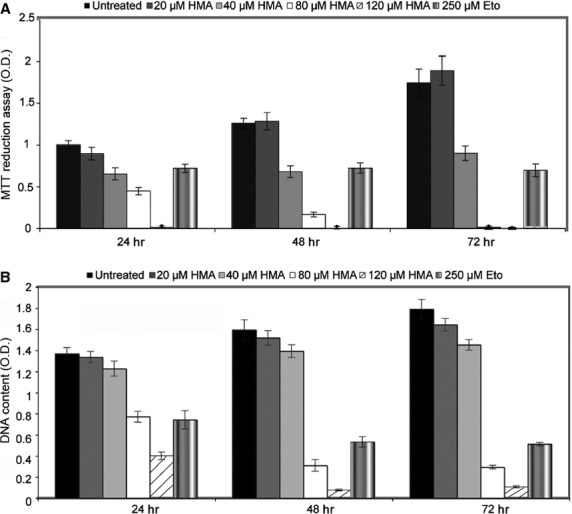
HMA effect on ARPE-19 cell viability and proliferation. ARPE-19 cells were treated with increasing concentrations of HMA (20–120 μM) for up to 72 hrs. Cell incubation with 250 μM etoposide for up to 72 hrs was used as an internal standard for caspase-dependent apoptosis inducers. (**A**) Cell viability was evaluated by the MTT assay after 24, 48 and 72 hrs of treatment. Results are expressed as the mean ± SD of three independent experiments. All measurements were significantly different as calculated using a one way anova test *P* < 0.05 (**B**) To monitor cell proliferation, cells were lysed in alkaline buffer and the released DNA was measured. Results are expressed as the mean ± SD of three independent experiments All measurements were significantly different as calculated using a one way anova test *P* < 0.05.

To test ARPE-19 cell response to HMA, we first measured cell viability by the MTT (3-(4,5-Dimethylthiazol-2-yl)-2,5-diphenyltetrazolium bromide) assay. Treatment of ARPE-19 cells with different HMA concentrations (20–120 μM) for increasing times (up to 72 hrs) revealed that HMA has a dose/time-dependent effect on ARPE-19 cell viability (Fig. 1A), with an IC50 ∼60 μM. This value is higher than the IC 50 for other cells (∼20 μM for BHK, HeLa and colon carcinoma cells, not shown). Etoposide (a well-known apoptosis inducer) did not affect ARPE-19 cell viability, possibly causing an arrest in cell proliferation, as suggested by the absorbance values at different time-points (Fig. 1A, Eto).

To evaluate the impact of HMA on cell proliferation, we measured the DNA released from total extracts after alkaline lysis; this parameter is proportional to the cell number [9]. This assay further confirmed the dose/time-dependent decrease in ARPE-19 cell number after HMA treatment (Fig. 1B). On the whole, these results indicated that HMA interferes with the proliferation and viability of ARPE-19 cells, triggering also cell death.

**Fig. 2:**
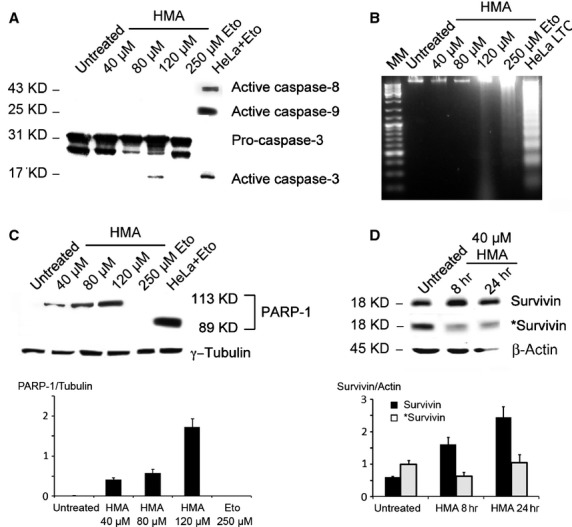
Analysis of caspase-dependent apoptosis in ARPE-19 cells treated with HMA. (**A**) ARPE-19 cells were treated with different HMA concentrations (40–120 μM) for 24 hrs and then analysed using Western blot. Etoposide (250 μM), administered for 24 hrs, was used as an internal standard. As a positive control for apoptosis, HeLa cells were treated with 100 μM etoposide for 3 hrs followed by 24 hrs of recovery in drug-free medium. The activation of caspases 3, 8 and 9 was investigated. Only caspase 3 was slightly activated in 120 μM HMA treated cells. (**B**) ARPE-19 cells were treated for 24 hrs with HMA (40–120 μM); 250 μM etoposide administered for 72 hrs was used as a pro-apoptotic drug. Long-term cultured (LTC) HeLa cells were used as a positive control for apoptosis. Nuclear DNA was extracted and loaded on a 1.8% agarose gel stained with ethidium bromide. No DNA degradation was visible in untreated cells or in cells treated with HMA 40 or 80 μM. A smear was observed in cells treated with 120 μM HMA and a faint ladder is seen in etoposide-treated ARPE-19 cells. (**C**) Upper panel. Western blot analysis of PARP-1 proteolysis was performed on untreated, HMA- or etoposide-treated ARPE-19 cells. HeLa cells treated with etoposide were used as a positive control. γ-Tubulin was used as a loading control. 113 kD: full length PARP-1; 89 kD: cleaved PARP-1. Lower panel shows a quantification of the expression of full length PARP-1 compared to γ-tubulin. At the used loading of proteins on the gel PARP-1 is not detectable in untreated or etoposide-treated cells, but its expression increases with HMA concentration. All the represented means are different from each other as calculated from a one way anova test (*P* < 0.0001). (**D**). Upper panel. Western blot analysis of survivin and its phosphorylated form (*) was performed on untreated ARPE-19 cells or cells treated for 8 or 24 hrs with 40 μM HMA. β-Actin was used as a loading control. The quantification of these experiments is seen in the lower panel, where the expression of survivin (black bars) or phosphorylated survivin (grey bars) is compared to the expression of β-actin. All the means are different from each other as calculated from a one way anova test (*P* < 0.0001 for survivin, *P* < 0.05 for phosphorylated survivin).

To verify if HMA cytotoxic effect was correlated to the apoptotic process, we evaluated initiator caspase status after drug treatment, and found no evidence of caspase-8, and -9 activation; moreover, effector caspase-3 showed a modest conversion into the active form only in cells treated with 120 μM HMA (Fig. 2A). Similar results were obtained with etoposide (Eto), thus confirming the intrinsic resistance of ARPE-19 cells to activate caspase-dependent apoptosis, as further supported by the absence of DNA ladder in HMA-treated cells (Fig. 2B). The analysis of PARP-1 proteolysis, which is a classical marker of caspase-dependent cell death [9], further confirmed that HMA did not induce classical apoptosis (Fig. 2C). To give some insights into the resistance of ARPE-19 to activate caspases, we investigated the expression of survivin, a major survival factor that blocks apoptosis through the inhibition of caspases. To our surprise, survivin, which is normally expressed in embryonic and cancer cells is highly expressed in ARPE-19 cells. There was an increase in surviving expression, as compared with the expression of actin (Fig. 2D). The ability of HMA to inhibit survivin activation by interfering with protein phosphorylation on Thr^34^ residue was also investigated. Results from immunoblotting experiments using a phosphospecific antibody indicated that the levels of the active, Thr^34^-phosphorylated form of survivin were reduced following treatment of ARPE-19 cells with HMA at both time-points (Fig. 2D), suggesting that this factor, even if present, is not protecting ARPE-19 cells from death.

**Fig. 3:**
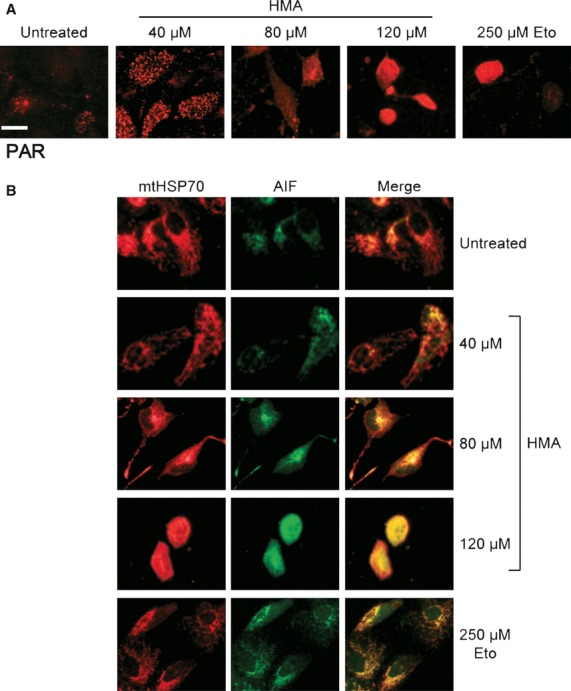
Indirect Immunofluorescence analysis of PARP-1 activity and AIF-dependent parthanatos. (**A**) ARPE-19 cells treated with either HMA or etoposide were probed with anti-PAR antibody to evaluate PAR synthesis. (**B**) Evaluation of AIF localization in HMA-treated ARPE-19 cells. Experiments were carried out with anti-mtHSP70 (to label mitochondria, red fluorescence) and anti-AIF (green fluorescence) antibodies. Scale bar 25 μm.

As mentioned above, no cleavage of PARP-1 was observed but, unexpectedly, HMA-treated cells showed increased levels of PARP-1 expression (Fig. 2C). To investigate if this event was correlated to increased PARP-1 activity, we performed immunofluorescence experiments using an anti- poly(ADP-ribose) (PAR) antibody (Fig. 3A). PAR synthesis, which is a stress response signal, appeared to be increased after HMA exposure. In fact, HMA stimulated PAR accumulation, visible as the appearance of speckled nuclei after 40 μM HMA treatment for 24 hrs. The treatment of cells with higher HMA concentrations (80 μM and 120 μM) or 250 μM etoposide revealed the appearance of brilliant nuclei (Fig. 3A).

Given that PAR synthesis is involved in the caspase-independent apoptotic pathway called parthanatos, driven by AIF (Apoptosis Inducing Factor) translocation from mitochondria to the nucleus [10], we investigated intracellular AIF localization. In untreated ARPE-19 cells, AIF co-localized with the mitochondrial marker mtHSP70 (Fig. 3B). The treatment with 40 μM and 80 μM HMA for 24 hrs did not promote a significant AIF translocation from mitochondria to the nucleus, which was visible in cells treated with 120 μM HMA, where cells became rounded and AIF appeared to move to the nucleus. These data suggest that the activation of this pathway occurs only at high HMA concentrations.

**Fig. 4:**
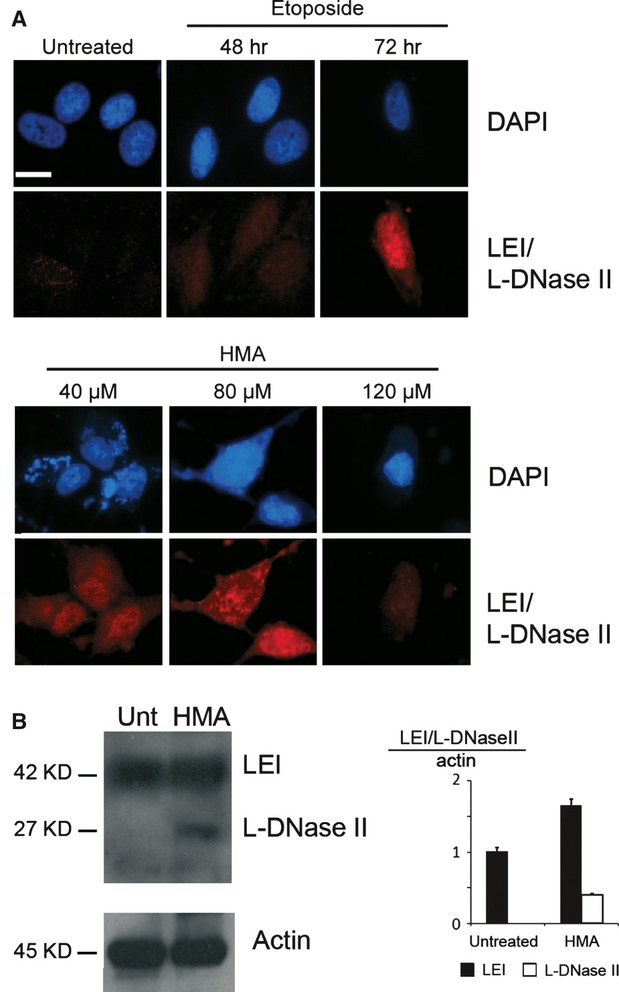
Analysis of LEI/L-DNase II in caspase-independent apoptosis. (**A**) Indirect immunofluorescence experiments were performed on ARPE-19 cells untreated or treated with HMA for 24 hrs at the indicated concentrations or with 250 μM etoposide for the indicated times. DAPI (blue) or anti-LEI/L-DNase II antibody (red) was used. Scale bar: 25 μm. (**B**) ARPE-19 cells were treated with 40 μM HMA for 24 hrs and analysed by Western blot using anti-LEI/L-DNase II antibody. β actin was used as loading control. Right panel shows the quantification of the LEI/L-DNase II western. Black bars represent LEI/actin ratio, white bar represents L-DNase II/actin ratio. There is an increase of LEI expression and of L-DNase II in treated cells. Means between treated and untreated cells are statistically different as compared by using *t*-test (*P* < 0.005 for LEI, *P* < 0.0001 for L-DNase II).

We investigated also a paradigm of apoptosis that involves leucocyte elastase inhibitor (LEI) conversion into its DNase active form, called L-DNase II [11] and that implies the activation of PARP-1. [12, 13] Immunofluorescence experiments revealed that in untreated cells, the labelling for LEI/L-DNase II proteins showed a low and diffuse pattern, while a treatment of 72 hrs with 250 μM etoposide induced the translocation of LEI inside the nucleus of ARPE-19 cells (Fig. 4A). In HMA-treated cells (40 μM), the nuclear fluorescence of LEI/L-DNase II supported its translocation inside the nucleus. Using higher drug concentration (80 μM), L-DNase-II aggregated into foci inside the nucleus; however, this phenomenon was no longer visible in cells treated with 120 μM HMA (Fig. 4A). The conversion, and therefore activation, of LEI into its L-DNase II form was further confirmed using Western blot analysis, which revealed the appearance of an additional immunoreactive band (corresponding to L-DNase II) after HMA treatment (Fig. 4B). This body of evidence indicates that the activation of L-DNase II, an endonuclease known to be active in apoptotic-like cell death, was involved in ARPE-19 cell demise.

**Fig. 5:**
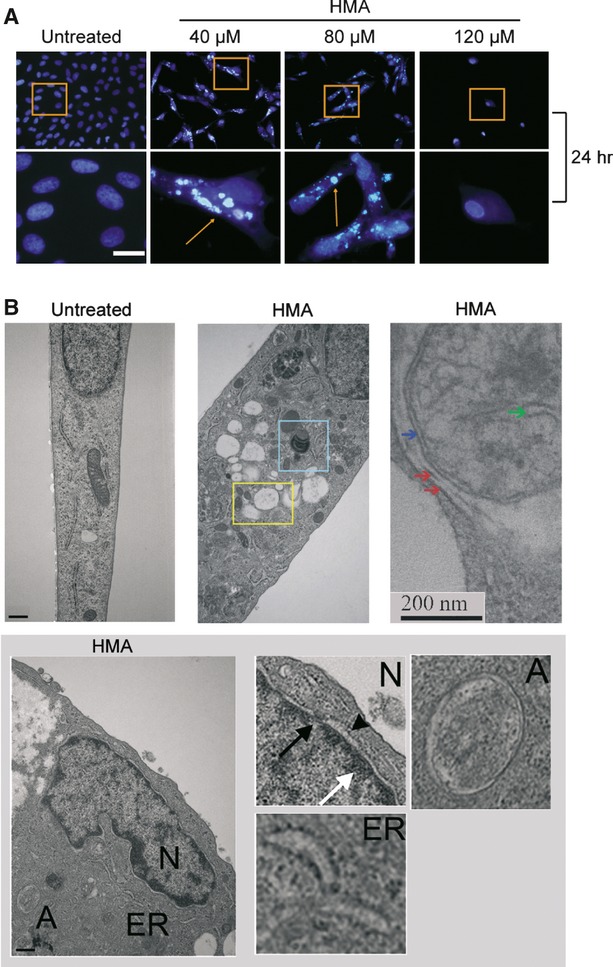
Morphological changes induced by HMA. (**A**) ARPE-19 cells were treated with the indicated HMA concentrations for 24 hrs, stained with DAPI and observed at the optical microscope. Fields in squares were magnified (lower panel). Scale bar 25 μm. (**B**) Electron microscope analysis was performed on ARPE-19 cells untreated (upper left panel) or treated with HMA (middle and right upper panels). In HMA-treated cells squares show vacuoles (middle upper panel). The left upper panel shows a high magnification image of an autophagosome (see the double membrane indicated by two red arrows) containing a mitochondrion. Blue arrow shows the double membrane of the mitochondrion. The green arrow indicates a mithocondrial creast. Bottom panel: Higher magnifications show the nucleus (N), endoplasmic reticulum (ER), autophagosomes (A). In (N), white arrow shows chromatin condensation, black arrow indicates the nuclear pore and arrow head the dilatation of the nuclear membrane. Scale bar: 0.5 μm.

As apoptosis displays very characteristic changes in nuclear morphology, we evaluate HMA effect on this parameter. To do this, we stained ARPE-19 cells with DAPI after 40, 80 and 120 μM HMA treatment for 24 hrs. ARPE-19 nuclei showed no signs of canonical apoptosis (*i.e*. nuclear shrinkage and apoptotic bodies); interestingly, the cytoplasm of treated cells displayed various fluorescent and brilliant dots (Fig. 5A).

To get inside the nature of this fluorescence, we analysed HMA spectral behaviours, providing the evidence that HMA emits blue light when irradiated with UV light [14], and that the compound is accumulated in these vesicles. Electron microscopy observation revealed that 40 μM HMA treatment for 24 hrs triggered the formation of several vacuole-like structures different in shape and content, being either filled with electron-dense material, likely of mitochondrial origin, or empty and surrounded by double membranes, suggestive of autophagosomes (Fig. 5B, upper panel). Moreover, HMA treatment induced nuclear as well as endoplasmic reticulum membrane dilatation and phase I chromatin condensation, indicating that HMA affected both organelles and nuclear integrity (Fig. 5B, lower panel).

### HMA induces autophagy in ARPE-19 cells

**Fig. 6:**
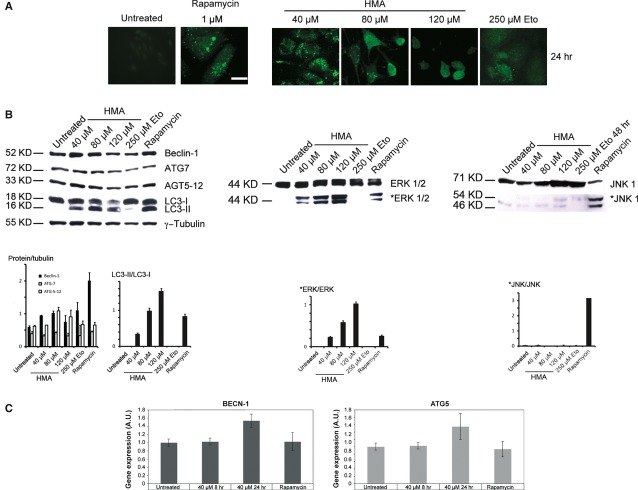
HMA induces autophagy. (**A**) ARPE 19 cells were untreated or treated with rapamycin for 2 hrs, with HMA or etoposide for 24 hrs and then probed with anti-LC3 antibody. Scale bar 25 μm. (**B**) ARPE-19 cells were treated as before and analysed by Western blot using anti-beclin 1, ATG-7, AGT5-12 and LC3 antibodies. γ tubulin was used as a loading control (left panel). On middle and right panels, cells were treated as before and analysed using anti-ERK and phospho-ERK (*) antibodies (middle panel) or with anti JNK1 or phosphorylated JNK1 (*) antibodies (right panel). Under the western images the quantification of the bands is reported showing a significant increase of Beclin 1 (Means are different from each other as calculated from a one-way anova test (*P* < 0.0001). ATG7 did not change (*P* = 0.165, one-way anova). ATG 12 is increased in treated samples (Means are different from each other as calculated from a-one way anova test (*P* < 0.05) The lipidated form of LC3 was also significantly increased as compared with the control (*P* < 0.0001) for all treatments. The same holds true for the phosphorylated form of ERK (*P* < 0.0001, except for etoposide, anova test). JNK was significantly increased at 120 μM HMA and in the presence of rapamycin (anova test *P* < 0.0001). (**C**) RT real-time PCR experiments were performed on untreated and HMA or rapamycin-treated ARPE-19 cells. HMA was used at 40 μM concentration for the indicated times, whereas rapamycin was used at 1 μM concentration for 4 hrs. GAPDH expression level was used as internal standard. Results are expressed as the mean ± SD of three independent experiments. Relative expression levels for each gene were calculated using the ΔΔCt method. Only the 40 μM HMA results are significantly different from the others *P* < 0.05.

To investigate the nature of vesicle-like structures, we monitored the status of LC3 protein, a specific marker for autophagy (Fig. 6A and B). In untreated ARPE-19 cells, LC3 labelling was low and cytoplasmic; in rapamycin-treated samples, which are representative of autophagy, several LC3-labelled dots corresponding to autophagosomes were detectable (Fig. 6A, left panel). These structures were visible in cells treated with 40 and 80 μM HMA for 24 hrs, while 120 μM treated cells showed a more diffused labelling. In fact, the punctuated labelling of LC3 is apparent from 2 hrs of treatment with HMA (data not shown). DMSO alone, the vehicle of HMA produced a labelling identical to the control. Interestingly, also 250 μM etoposide caused the appearance of few autophagosomes in ARPE-19 cells (Fig. 6A, right panel).

The analysis of LC3 status using Western blot further confirmed that HMA induces autophagy, as demonstrated by the appearance of LC3-II protein, the lipidated form of LC3 (Fig. 6B, left panel). In etoposide-treated cells, a modest amount of LC3-II was also present, suggesting that autophagy activation is a general feature of ARPE-19 cells.

We further investigated the expression of different proteins involved in the autophagic process. Western blot analysis revealed that Beclin-1, one of the most important autophagy regulators, was up-regulated by 40 and 80 μM HMA; the same effect was recorded for ATG5/12 (Fig. 6B, left panel). By contrast, no significant changes in ATG7 expression were detected. Remarkably, HMA-induced autophagy was found to be regulated by the MAPK pathway, as supported by the phosphorylation of ERK 1/2 proteins, which activation was sustained at least up to 24 hrs (Fig. 6B, central panel). On the contrary, JNK, which is known to be involved in several paradigms of autophagy, was poorly activated by HMA (Fig 6B, right panel).

Finally, up-regulation of Beclin-1 and ATG5 protein levels was confirmed by RT real-time PCR. As shown in Figure 6C, 40 μM HMA induced an up-regulation of BECN-1 and ATG5 gene expression. In fact, although an 8-hr treatment had no effect on BECN-1 and ATG5 gene transcription (1.03 and 1.02 A.U. respectively), a 24 hrs treatment up-regulated BECN-1 (1.53) and ATG5 (1.55) gene transcription.

### Role of autophagy in HMA-treated ARPE-19 cells

**Fig. 7:**
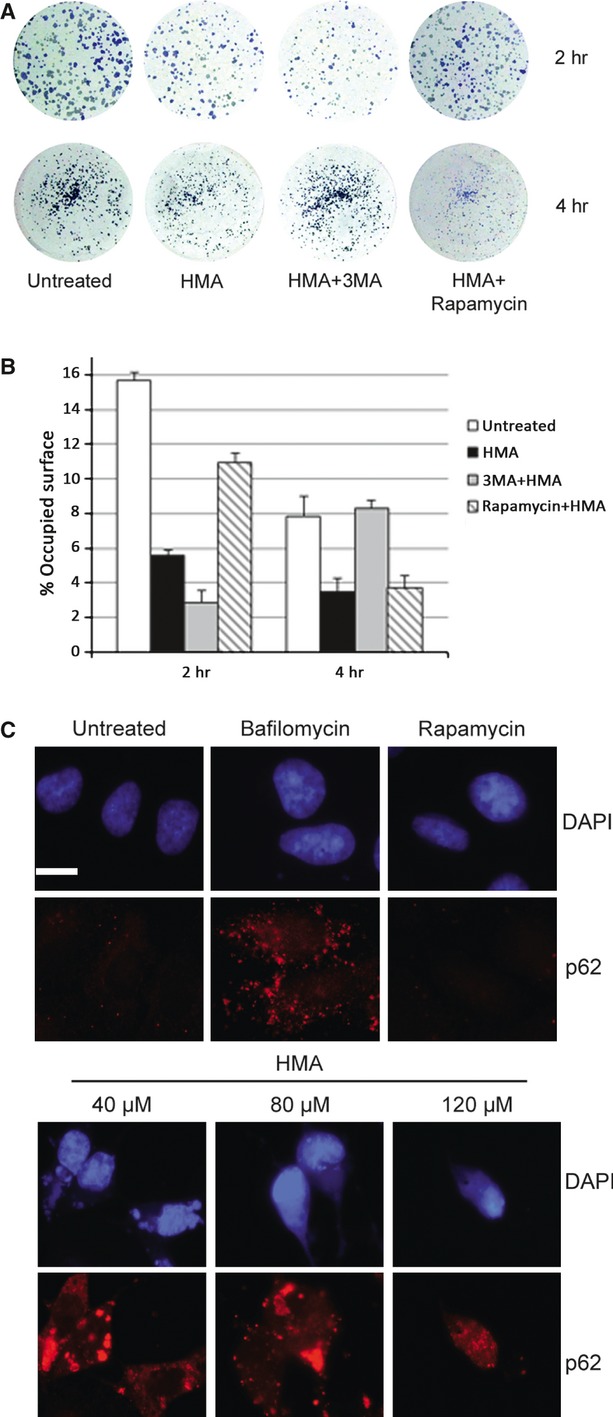
Role of autophagy in HMA response. (**A**) ARPE-19 cells were treated with HMA in the absence or presence of 1 μM rapamycin or 5 mM 3-MA. After 2 or 4 hrs of treatment, cells were trypsinized and counted. A total of 1000 cells were seeded into 6-well plates. Seven days after seeding, cells were stained with cresyl violet. Controls using rapamycin or 3-MA alone have show no difference with the control (not shown) (**B**) Using image analysis, the surface covered by the cells was measured and plotted. For 2 hrs of treatment all means were significantly different (*P* < 0.001) for 4 hrs no significant difference was found between control and HMA+3-MA treated cells (student test *P* = 0.73) or between HMA and HMA + rapamycin-treated cells (*P* = 0.87). The treatment of cells with rapamicin or 3-MA alone did not have any effect on cell survival (not shown). (**C) **HMA-treated ARPE-19 cells, at different concentrations for 24 hrs, were stained with DAPI (upper panel) and anti-p62 antibody (lower panel). HMA-treated samples were compared to 50 nM bafylomicin-treated (representative of lysosome-autophagosome fusion failure) or 1 μM rapamycin (representative of autophagy)-treated cells. Scale bar 25 μm.

To depict the exact role of autophagy in HMA-treated ARPE-19 cells, we used either rapamycin (autophagy inducer) or 3-methyladenine (3-MA), an autophagy inhibitor that blocks autophagosome fusion with lysosome, in combination with HMA or alone. The clonogenic assay performed in ARPE-19 cells treated with HMA for 24 hrs showed low survival and no difference between untreated and treated cells (data not shown). However, a short incubation with HMA (2 hrs) alone or combined with either 3-MA or rapamycin, revealed that inhibition of autophagy by 3-MA increased cell death, while rapamycin had the opposite effect (Fig. 7A and B). Surprisingly, after further 2 hrs of HMA exposure the situation was inversed, 3-MA bringing protection, rapamycin increasing cell death (Fig. 7A and B). These results suggest that early activation of autophagy protects cells from HMA insult, but after few hours a prolonged activation of the autophagic process might be deleterious.

The effect of HMA treatment in ARPE-19 cells on autophagic flux was evaluated by indirect immunofluorescence focusing on p62 protein localization. p62 is involved in the recognition and delivery to autophagosomes of proteins to be degraded by the autophagic machinery; its expression levels are a specific marker for autophagic flux [15]. In untreated cells, p62 showed a diffuse intracellular pattern, as in the case of rapamycin-induced autophagy (Fig. 7C). HMA triggered the re-localization of p62 giving rise to a punctate cytoplasmic pattern, as in the case of bafilomycin (an inhibitor of lysosomal ATPase, which blocks autophagosome fusion with lysosome) -treated cells, where several puncta appeared inside the cytosol. Interestingly, in HMA-treated cells p62 formed brilliant foci, possibly because of high protein damage caused by HMA in specific cellular districts.

**Fig. 8:**
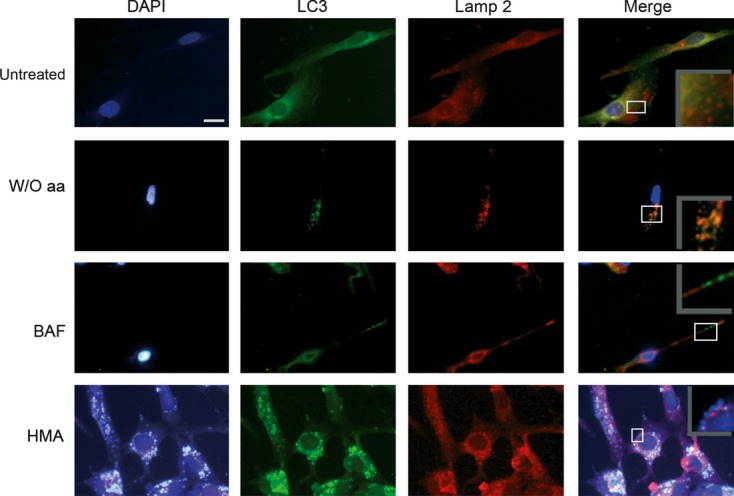
Lysosome, autophagosome fusion in HMA treated cells. ARPE cells were cultured in normal medium (untreated), in a complete medium without aminoacids (to induce autophagy), in the presence of 50 nM bafilomycin (to inhibit the lysosomal proton pump) or 40 μM HMA. Immunofluorescence was performed using DAPI staining (blue), anti-LC3 (green) and anti-LAMP 2 (red) antibody. Insets in the ‘merge’ panel represent a zoomed region of the merged picture. In ‘untreated cells’ only lysosomes (red) show a punctuate staining. In the absence of amino acids (W/O aa) red and green stained (lysosomes and autophagosomes respectively) merged. These stains are not overlapped in bafilomycin-treated cells neither in HMA-treated cells. To note: given that HMA is fluorescent in UV conditions autophagosomes appear white in the merged pictures, lysosomes are red. The nucleus stained with DAPI is less fluorescent than the vesicles and appears artificially unlabelled. Scale bar: 25 μM.

To investigate the fusion between autophagosomes and lysosomes, we analysed specific proteins resident in these two organelles, namely, LC3 in the autophagosomes and LAMP-2 in the lysosomes (Fig. 8). Untreated cells show only a red puncture labelling corresponding to lysosomes (Fig. 8 upper row, merge, inset). In ARPE-19 cells driven to autophagy by aminoacid depletion (W/O aa), a complete overlapping of both proteins was visualized (see merge and inset). This was not the case for bafilomycin- and HMA-treated cells, in which the proteins did not colocalize (see Fig. 8, BAF and HMA rows), supporting the hypothesis that HMA inhibits the fusion of autophagosome with lysosome. It is worth noting that in HMA-treated cells the DAPI staining labels mainly the vesicles. This is because of the autofluorescence of HMA [15].

## Discussion

The aim of the present study was to exploit an *in vitro* model system representative of the *in vivo* dangerous milieu of retinal cells, *i.e*. where cells have to face a lot of exogenous insults, causing often adverse growth conditions. Among the deleterious situations recorded *in vivo*, changes in the pH occur, which can be reproduced *in vitro* by the use of HMA, a NHE inhibitor belonging to the amiloride family, which is a potent inhibitor of Na^+^/H^+^ exchangers through the plasma membrane, able to induce apoptosis in most cells [7].

Adult retinal pigmented epithelial-19 cells proved to be extremely resistant to HMA treatment, and a similar cell response was recorded also for etoposide, forcing the concept, supported by other's results, that these cells are very resistant to stress [16]. It is worth noting that etoposide activates classical (caspase-dependent) apoptosis in most cells, while HMA triggers caspase-independent, apoptotic-like cell death [7].

We show here that ARPE-19 cells overcome cell death by activating autophagy. In the first 2 hrs of HMA exposure, autophagy is efficient in protecting cells from death. Thereafter, autophagy is impaired, as indicated by a p62 accumulation, and this protective mechanism becomes the executioner of cell death. This switch in autophagy role as a function of time for a single paradigm is shown here for the first time.

Our results show that, depending on concentration, HMA has two different effects. Up to 80 μM, an autophagic response is observed, but at higher concentrations (120 μM) a modest activation of caspase-3, AIF translocation and LEI conversion into L-DNase II have been detected, suggesting that multiple cell death pathways are simultaneously triggered as result of this extremely high dose.

Given that the apoptotic response (caspase activation; Fig. 2A) occurs at very high HMA concentration, we focused our attention on the events occurring at lower concentrations, possibly involved in cell resistance. The main response of these cells to HMA concentration ranging from 20 to 80 μM is the activation of the autophagic process that, likely, protects ARPE-19 cells (Fig. 6). Actually, the clonogenic test showed that this is true in the first 2 hrs of HMA treatment (Fig. 7A). In this condition, as expected, the activation of autophagy by rapamycin increases cell viability, while inhibition by 3-MA increases cell death. This protection is probably a result of the sequestration of damaged organelles/proteins into autophagosomes (Fig. 5). It is worth noting that in ARPE-19 cells we detected also HMA in autophagosomes, indicating that the compound itself is sequestered in the vesicles [14] (Fig. 5A). Western blot analysis of molecular actors of autophagy supports the activation of this process; in fact, LC3 is transformed in LC3-II, this event being a marker of autophagy; furthermore, *in situ* experiments confirm the formation of autophagosomes after HMA treatment (Fig. 6). Moreover, overexpression of beclin-1 suggests that this HMA-triggered pathway of autophagy is beclin-dependent. Stress kinases ERK 1/2 and JNK 1 are also activated (even if JNK1 activation is very low if related to rapamycin-treated cells; Fig. 6B). In this regard, it has been shown that a transient activation of ERK may induce autophagy, although its sustained activation might be deleterious for a correct autophagy execution [17] by inhibiting autophagosome maturation, giving rise to giant autophagosomes unable to fuse with lysosomes, thus blocking the final step of autophagy. It is possible that, in our biological system, ERK protein is at least in part involved in the formation of large autophagosomes as well as in the possible inhibition of their maturation, as indicated by p62 accumulation within the cytosol (Fig. 7B) and the lack of fusion between phagosomes and lysosomes (Fig. 8).

No signs of caspase-dependent cell death have been detected after HMA treatment, even if different features of cellular damage such as nuclear and endoplasmic reticulum membrane dilatation and chromatin condensation have been observed using electron microscope analysis (Fig. 5B), confirming the notion that the ARPE-19 cell line is extremely refractory to caspase-dependent cell death induction.

It is interesting to note that PARP-1, a ‘guardian of the genome’ with a crucial role in maintaining DNA integrity under stress condition [18] is overexpressed (Fig. 2C) and hyperactivated (Fig. 3A) after HMA treatment. At the same time, we found the activation of L-DNase II (Fig. 4), an endonuclease [12, 13] which is in charge of DNA degradation to promote the dismantling of the nucleus of the dying cell and could mediate PARP-1 activation, as we already reported [12]. In our study, the mechanism of activation of PARP-1 seems quite different from the mechanism described by Muñoz-Gamez *et al*. [19], who showed that PARP-1 is activated by the damage induced in DNA and autophagy is abolished in PARP-1^−/−^ cells, suggesting a role of this enzyme in the regulation of the autophagic process [20]. Here, PARP-1 activation might be just a consequence of L-DNase II activity, which is not very high because no DNA degradation is visible in agarose gels.

PARP-1 activation and consequent PAR synthesis have been related to a caspase-independent cell death pathway called parthanatos, in which PAR stimulates AIF translocation from mitochondria to the nucleus, where it induces DNA condensation and cell death (Fig. 3B) [10]. This could be the case for the cell death observed after 120-μM HMA treatment, in agreement with previous data obtained in our laboratory showing that caspase-independent pathways are preferentially activated in ARPE-19 cells [21].

Taken together, our data allow the demonstration that under cellular stress ARPE-19 respond by triggering autophagy. This response is not completely efficient in protecting cells because of the pleiotropic effects of HMA, possibly unrelated, being able to induce autophagy, on the one hand, and to block it, on the other hand [22]. In this scenario, sustained autophagy activation has a deleterious effect on cell viability and its forced and prolonged activation could switch its role from cell rescue to PCD type II. Beyond the time-dependent role of autophagy in manipulating cell death, our data support the idea that autophagy can be a general response after drug treatment. Actually, this behaviour is also observed at a lesser extent with etoposide, suggesting that induction of autophagy might be a general response of RPE to stress and the most important pathway involved in cell resistance to adverse conditions and toxic stimuli. Experiments in progress are devoted to extend our analysis to *in vivo* pathological conditions.

It is worth noting that lysosomes play a major role in RPE physiology because of their involvement in the management of photoreceptor waste. Actually, it has been shown that *N*-retynildiene—*N*-retynilethanolamine, a lipofuscin pigment causes inhibition of the proton pump and lysosomal disfunction [23].This is also the case for the lysomotropic agent chloroquine [24]. In addition, mice deficient in Nrf2, an antioxidant gene develop deregulated autophagy and cardinal features of age-related macular degeneration, including drusen formation [25]. Thus, the investigation of the regulation of the autophagic process in RPE cells could be a clue point for the understanding of the physiopathology of age-related macular degeneration and the development of new therapeutic approaches.
